# The Association Between Isometric Shoulder Strength and Sports Performances in University Soccer Players: A Cross-Sectional Study

**DOI:** 10.7759/cureus.72041

**Published:** 2024-10-21

**Authors:** Ali I Khan, Sumbul Ansari, Zahid Khan, Shahid Raza

**Affiliations:** 1 Center for Physiotherapy and Rehabilitation Sciences, Jamia Millia Islamia, New Delhi, IND; 2 Department of Physiotherapy, School of Medical and Allied Health Sciences, Galgotias University, Greater Noida, IND; 3 Department of Acute Medicine, Mid and South Essex NHS Foundation Trust, Southend-on-Sea, GBR; 4 Department of Cardiology, Barts Heart Centre, London, GBR; 5 Department of Cardiology and General Medicine, Barking, Havering and Redbridge University Hospitals NHS Trust, London, GBR; 6 Department of Cardiology, Royal Free Hospital, London, GBR

**Keywords:** adolescent soccer players, agility athletes, agility training, kinesthetic balance agility, lower-body strength, soccer player, sports physiotherapist, sports rehabilitation, sprint performance, strength training

## Abstract

Background

Soccer, a globally popular sport, demands a complex interplay between physical attributes, including speed, agility, power, and endurance. Although lower-body strength and power are often emphasized, the role of upper-body strength, particularly shoulder strength, remains less explored. Given the importance of upper-body movements in activities such as heading, shooting, and defending, understanding the relationship between shoulder strength and soccer performance is crucial.

Aims

This study aimed to explore any possible correlation between isometric shoulder muscle strength (flexors and extensors) and sports performance (sprint and agility) and to evaluate whether isometric shoulder strength is associated with sports performance in university-level soccer players.

Methods

A total of 35 male amateur soccer players were recruited, who underwent demographic measurements such as age, height, weight, and body mass index (BMI), and were then subjected to isometric strength assessment of the shoulder flexors and extensors using a handheld dynamometer (HHD). Subsequently, the players' sprint and agility performances were recorded. Appropriate statistical tests were performed on the obtained data.

Results

The findings revealed a significant negative correlation between shoulder flexor strength and sprinting (r=-0.707, p<0.01) and between shoulder extensor strength and sprinting (r=-0.611, p<0.01). There was no significant correlation between shoulder flexor strength and agility (r=-0.121, p=0.48) or between shoulder extensor strength and agility (r=-0.212, p=0.22). Multiple linear regression analysis revealed that only shoulder flexor strength (β=-0.688, t=-2.651, p=0.01) was found to have statistically significant relationships with sprint performance, explaining 50% of the variance in sprint performance.

Conclusions

The present study found a negative bidirectional relationship between shoulder muscle strength and sprint performance. Shoulder flexor strength explained 50% of the variance in sprinting performance. This information is useful for physiotherapists, coaches, and trainers to focus on strengthening the shoulder musculature to improve performance.

## Introduction

Soccer is the most popular sport in the world, played by males and females, children, and adults at various levels of competition [1]. Soccer performance is determined by a variety of elements, including technical, biomechanical, tactical, mental, and physiological aspects. During the game, soccer players must leap, kick, tackle, pivot, run, change tempo, and sustain powerful contractions to maintain balance and control the ball under defensive pressure [2]. Increased available force (from essential muscle contractions), as well as acceleration and speed in soccer abilities such as turning, sprinting, and changing pace, may improve performance [3].

During a match, both male and female soccer players run approximately 9-12 km [4]. High-intensity running or sprinting accounts for 8%-12% of the total [4]. Compared to other play positions, wide midfielders and external defenders have higher running and sprinting efforts [5]. Peak sprint speed among soccer players has been reported to be 31-32 km/hour [6]. Efficient sprinters have an arm swing that begins from the shoulder and has the same amplitude of flexion and extension as the flexion and extension at the ipsilateral shoulder and hip [7]. If the arms were missing but the trunk was still present, a person could run but only at a much slower pace, because the trunk's moment of inertia about the vertical axis would be too small to generate the angular momentum required to balance the legs at these speeds of running, even if it vigorously twisted back and forth [8]. The ability to change directions quickly is also a crucial factor in good performance in sports, where cutting moves are regularly used [9]. During a soccer game, players can make up to 800 cutting actions [10]. As a result, the capacity to change direction quickly is based on various forms of strength [11,12]. However, the effects of arm movements and subsequent strength are not well-understood [8,13].

In addition, several researches have only shown a correlation between short sprint performance and lower-body strength [14-18]. Maximal strength and power are well-connected with sprint ability in the majority of sports studied [19]. However, the strength of these associations is modified by the group and sports studied. Previous research has found that strength has a considerable influence on upper-body performance in top kayakers [20,21], wheelchair racers [22], surfers [23], and luge athletes [24,25]. The relationship between upper limb muscular strength (shoulder flexor and extensor strength) and soccer-related performance, such as speed and agility, remains unknown. Given the importance of upper-body movements in soccer activities, such as heading, shooting, and defending, understanding the relationship between shoulder strength and performance outcomes is crucial. The current study sought to determine whether there is a relationship between isometric shoulder muscle strength (flexors and extensors) and soccer-related performance indicators (sprinting and agility). We hypothesized that there would be a significant correlation between isometric shoulder muscle strength (flexors and extensors) and sports performance (sprint and agility), and isometric shoulder strength could predict sports performance in university-level soccer players.

## Materials and methods

Objectives

The objectives of this study were to explore the possible association between sprint and shoulder flexor and extensor muscles in young university football players. Age and body mass index (BMI) were the likely confounders in this study.

Ethical considerations

This study was approved by the Institutional Ethics Committee of Jamia Millia Islamia (approval number: 31/10/185/JMI/IEC/2018) and was conducted in accordance with the Helsinki Declaration of 1964 and its later amendments. All players were informed about the study procedure, and written informed consent was obtained from all individual participants included in the study.

Study design and setting

The present study was a correlational study. The study took place at the Nawab Mansur Ali Khan (MAK) Pataudi Sports Complex, Jamia Millia Islamia, and the Laboratory of Biomechanics of the Center for Physiotherapy and Rehabilitation Sciences, Jamia Millia Islamia, New Delhi, India.

Sample size

The sample comprised university-level amateur soccer players. The number of subjects was determined using the software G*Power 3.1.9.2 (Heinrich-Heine-Universität Düsseldorf, Düsseldorf, Germany), using data of 20 m sprint and lower limb strength variables from a previous study on the relationship between strength, speed, and change of direction performance in field hockey players based on an effect size of 0.62, alpha level of 0.01, and power (1-beta) of 0.90 [23]. Based on these estimates, a sample of 35 players was found to be necessary.

Eligibility criterion

To be included in the study, the soccer player had to be a university-level player, actively involved in soccer sports activity and training for the past year, aged 18-28 years, free of any ongoing medical condition, and able to comprehend English. Players with any shoulder, hip, knee, or ankle surgery; signs and symptoms of upper or lower limb neurological compression; low back pain or any injury; or a history of cardiopulmonary conditions were excluded. Based on these criteria, 35 university-level soccer players were recruited by convenience sampling from Nawab MAK Pataudi Sports Complex, Jamia Millia Islamia, New Delhi, India.

Study procedures

All the experimental procedures were performed at the Laboratory of Biomechanics of the Center for Physiotherapy and Rehabilitation Sciences, Jamia Millia Islamia, and at the Nawab MAK Pataudi Sports Complex, Jamia Millia Islamia. After recording the demographic measurements such as age, height, weight, and body mass index (BMI), the players were subjected to an assessment of the isometric strength of the shoulder flexors and extensors. Subsequently, the players' sprint and agility performances were recorded. The order of assessment was kept the same for all players.

Isometric strength

The isometric strength of the shoulder flexors and extensors was measured using a Lafayette® Hand-Held Dynamometer Model 01165A (Lafayette Instrument Company, Lafayette, IN). It is a portable microprocessor-controlled equipment used to quantify isometric muscular strength. The measurement range of the instrument was 1335 N. A handheld dynamometer (HHD) is a reliable instrument for measuring muscle strength [25]. A make test was used to assess the maximum isometric strength [25]. The evaluator kept the dynamometer stationary while the player exerted maximum strength [25,26]. For all assessments, the players were instructed to execute the maximal isometric strength on the dynamometer while the evaluator encouraged them with a standardized phrase, "harder, harder, and harder"; before real testing, a familiarization session was conducted [23]. The player completed three maximum strength trials, and the average of the maximal strength recordings was used for analysis (sum of maximal strength trials/number of repetitions). Each trial was separated by one minute of rest. The maximal isometric strength was recorded by the device during the test in Newton [26].

To assess the isometric strength of the shoulder flexors, the player assumed a neutral sitting position with the feet on the ground with 90° shoulder flexion, 90° elbow flexion, and supinated forearm [23]. The HHD was placed perpendicular to the anterior surface of the distal third of the arm [23]. For the assessment of the isometric strength of the shoulder extensors, the player assumed a sitting position with the upper limbs by the side and the elbow extended [27]. The HHD was placed on the posterior aspect of the humerus, just proximal to the elbow [27]. The therapist stood posterior to the player in a stride-standing position and applied force in a posterior-to-anterior direction perpendicular to the limb [27]. Each player was asked to resist the movement.

Sprint

The 20 m sprint test was used to measure the sprint time. The 20 m sprint performance is considered a relevant performance parameter important for success in all sports involving sprints [28,29]. The players were instructed to warm up before the commencement of the test. Each player completed a minimum of three sprints, each separated by a 2-3-minute rest. The average of the three trials was used for the analysis [30]. A stopwatch was used to record the time.

Agility

The Illinois agility test was used to evaluate the agility of players. The test was set up with four cones to form an agility area [31]. On command, the player sprinted 9.20 m, turned, and returned to the starting line [31]. After returning to the starting line, the player swerves in and out of four markers, completing two 9.20 m sprints to complete the agility course [32]. The players performed three trials, and the average of the three trials was used for the analysis. A stopwatch was used to record the time.

Statistical analysis

All data were analyzed using the SPSS software (version 17.0, SPSS Inc., Chicago, IL). The normality of the data was checked using the Shapiro-Wilk test, and data that showed non-normal distribution were log-transformed for further analysis. Correlation analysis was performed using the bivariate model, and Pearson's correlation coefficient was calculated. The correlation coefficients ranging from 0.70 to 1.0 were regarded as strong, those between 0.6 and 0.4 as moderate, and those from 0.3 to 0 as weak [33,34]. It is worth mentioning that these can only be used to demonstrate possible association. Multiple linear regressions were used to identify whether sports performance (sprint and/or agility) was associated with shoulder flexor and extensor strength. Age and body mass index (BMI) were also used for multiple regression, along with shoulder flexor and extensor strength. Statistical significance was set at p<0.05.

## Results

**Figure 1:**
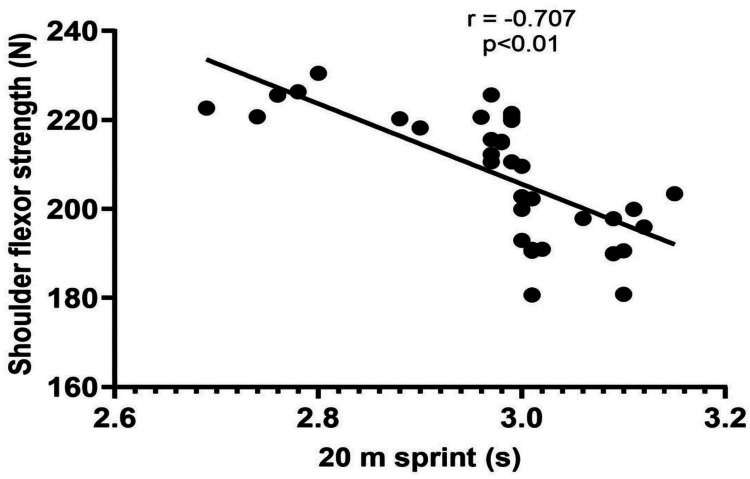
Relationship between isometric shoulder flexor strength and 20 m sprint performance

**Figure 2:**
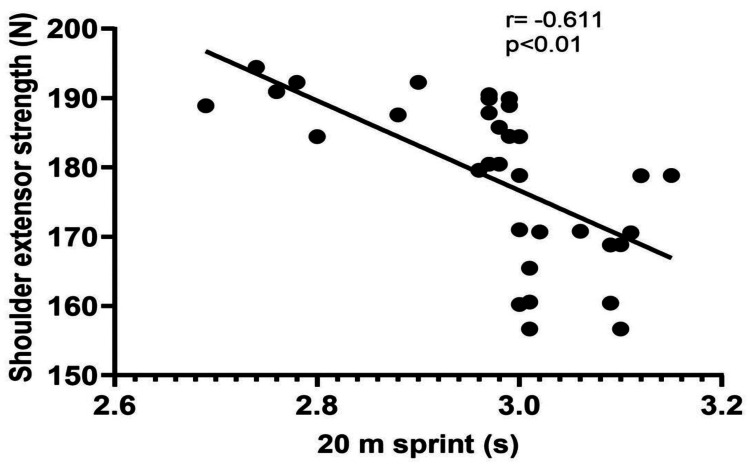
Relationship between isometric shoulder extensor strength and 20 m sprint performance

**Figure 3:**
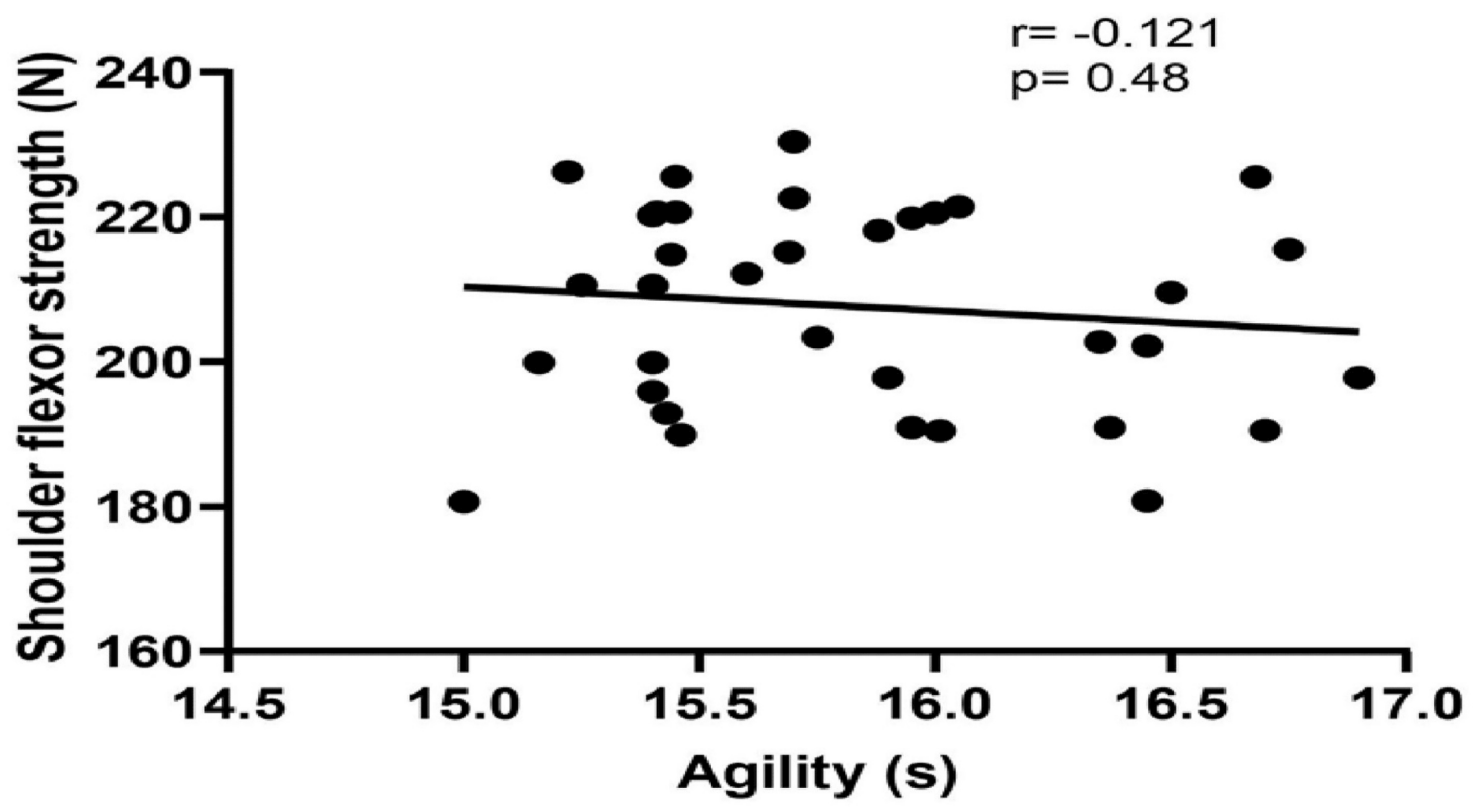
Relationship between isometric shoulder flexor strength and agility

**Figure 4:**
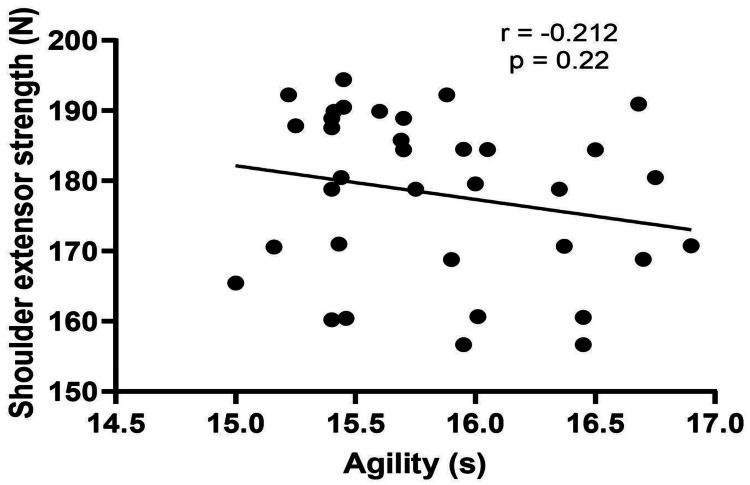
Relationship between isometric shoulder extensor strength and agility

**Figure 5:**
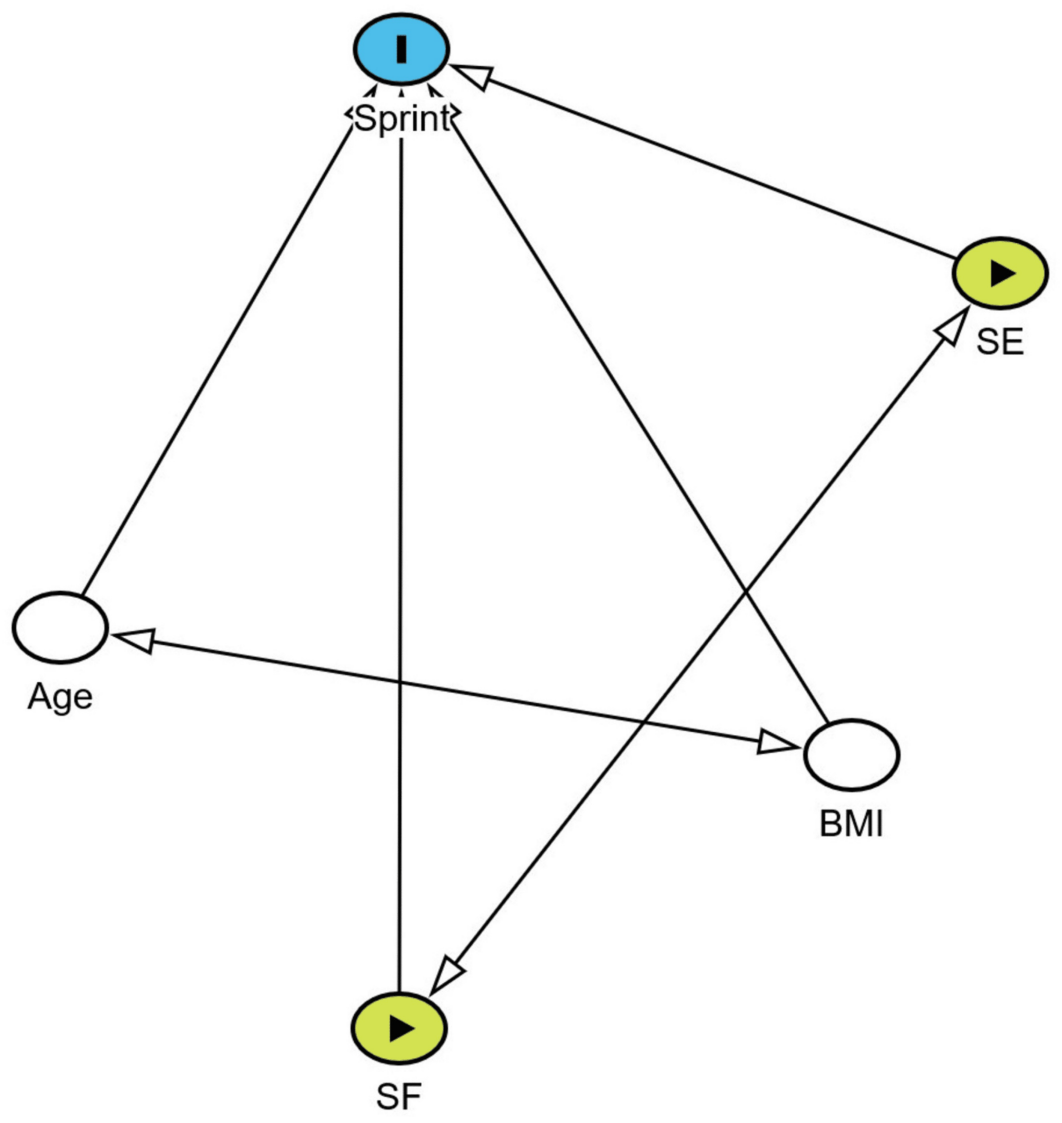
DAGitty graph adjusted for age and BMI showing the association between correlation exposure variables and the outcome variable BMI, body mass index; SF, shoulder flexors; SE, shoulder extensors

The demographic data of the players (age, height, weight, and BMI) are shown in Table 1. The results of the correlation analysis showed a significant strong correlation of 20 m sprint performance with shoulder flexor strength (p<0.01) and a significant moderate correlation with shoulder extensor strength (p<0.01) (Table 2, Figure 1, and Figure 2). However, there was no significant correlation between agility and the shoulder flexor or extensor muscle strength (p>0.05) (Table 2, Figure 3, and Figure 4). The average age of the study participants was 21.43±2.76 years, and the average weight was 62.17±7.10 kg. The average height was 169.00±6.14 cm. Pearson's correlation coefficient values for shoulder flexor and extensor strength were -0.707 and -0.611, respectively. The p-value for both groups was <0.01. We also created directed acyclic graphs to explore the correlation between the exposure and outcome variables by adjusting the confounders such as age and body mass index (BMI) as shown in Figure 5.

**Table 1:** Descriptive statistics of the players SD: standard deviation

Variables	Mean±SD
Age (years)	21.43±2.76
Height (cm)	169.00±6.14
Weight (kg)	62.17±7.10
BMI (kg/m^2^)	21.74±1.91
Shoulder flexor strength (N)	207.65±14.04
Shoulder extensor strength (N)	178.14±11.66
Sprint (seconds)	2.97±0.10
Agility (seconds)	15.83±0.51

**Table 2:** The value of Pearson's correlation coefficient (r) and significance level (p-value) for respective variables *Significant value

Variable	Statistical test/value	Shoulder flexor strength (N)	Shoulder extensor strength (N)
Sprint time (seconds)	Pearson's correlation	-0.707	-0.611
	p-value	<0.01*	<0.01*
Agility (seconds)	Pearson's correlation	-0.121	-0.212
	p-value	0.48	0.22

A multiple linear regression was performed to explore the association between shoulder strength including flexors and extensors, age, and BMI with sprint performance. The overall regression was found to be statistically significant, with an R-squared value of 0.50, F (4,30) of 7.51, and p-value of <0.001, indicating that the model explains 50% of the variance in sprint performance. From these associations, only the shoulder flexor strength was found to have a statistically significant relationship with the sprint performance (β=-0.688, t=-2.651, p=0.01). Shoulder extensor strength (β=-0.01, t=-0.07, p=0.94), age (β=-0.02, t=-0.14, p=0.88), and BMI (β=0.01, t=0.13, p=0.89) were not found to have statistically significant relationships with the sprint performance (Table 3).

**Table 3:** The regression analysis examining the relationship between the predictor variables with sprint performance *Statistically significant SE, standard error; LB, lower bound; UB, upper bound

Model (sprint)	Unstandardized coefficients		Standardized coefficients	t	p	95% CI for B	
	Beta	SE	Beta			LB	UB
Flexor strength (N)	-0.005	0.002	-0.688	-2.651	0.01*	-0.010	-0.001
Extensor strength (N)	0.000	0.002	-0.019	-0.072	0.94	-0.005	0.005
Age (years)	-0.001	0.005	-0.020	-0.147	0.88	-0.012	0.010
BMI (kg/m^2^)	0.001	0.008	0.019	0.137	0.891	-0.015	0.017

The main findings of the present study were as follows: (1) a significant strong negative correlation between shoulder flexor strength and sprint, (2) a significant moderate negative correlation between shoulder extensor strength and sprint, (3) no significant correlation between shoulder flexor strength and agility, (4) no significant correlation between shoulder extensor strength and agility, and (5) a multiple linear regression analysis revealed that only shoulder flexor strength significantly had positive correlation with sprint performance and the model explained 50% of the variance in sprint performance.

## Discussion

Sprint and shoulder flexor and extensor strength

According to the findings of our study, sprint performance and the strength of the shoulder flexors and extensors have a bidirectional relationship and move in the opposite direction. This typically means that if one increases, the other decreases. One of the most important soccer-related performances is sprinting, which is a complicated activity that requires full-body participation. Although lower limb strength is the most important predictor of sprint performance, the contribution of trunk and upper limb muscles cannot be overlooked [35,36]. Several researchers have examined the link between lower-body strength and sprint performance, and results have shown that stronger athletes perform better in sprints [12,15,36]. This may be explained by the fact that peak ground response forces and impulses are important predictors of sprint performance [37-40].

According to one study, arm swing is a characteristic of sprint running, with the arms functioning in a contralateral manner and the legs driving the body in a horizontal direction [41]. Arm-leg motions must be synchronized to achieve high acceleration and maximum speed. Another study showed that the arm-swinging movement increases the velocity by creating an enhanced push in the direction of advancement, which is especially important during the start and acceleration phases [42]. According to Miller et al., inhibiting arm swing can modify numerous lower limb biomechanics and kinematics [43]. According to one study, running (7-12 km/hour) boosts the activation of shoulder muscles 2-3 times more than walking at 5 km/hour [44]. Research on healthy females using electromyography found that active upper limb effort enhanced muscle activity in the passive lower limbs relative to passive upper limb effort [45]. Similarly, higher muscle activity in the passive upper limbs occurred during active lower limb effort than during passive lower limb effort, indicating a bidirectional impact [45]. Another study concluded that if the arms were absent but the trunk was still present, a person could run but only at a substantially decreased speed, because the trunk's moment of inertia about the vertical axis would be too minor to generate the angular momentum needed to balance the legs at these speeds of running, even if it vigorously twisted back and forth [46]. Furthermore, the arms can contribute up to 10% of the total vertical propulsive forces that an athlete can apply to the ground, emphasizing the need for effective arm motion [46-48]. In a study by Macadam et al., the body is upright during the maximal velocity phase of sprint running. The upper limb contributes to the overall vertical propulsive force delivered to the ground during sprinting, as in soccer [7]. A successful arm swing begins at the shoulder and includes flexion and extension movements corresponding to flexion and extension movements at the ipsilateral shoulder and hip [7]. In addition, Macadam et al. observed that the arms appear to counteract the rotational momentum of the legs while running, implying that the arms may also play an important role during the sprint start and early acceleration phases [7].

Because acceleration begins with the upper body, the soccer player must build upper-body strength to get off to a solid start. Furthermore, the upper-body strength and torso assist the sprinter in maintaining proper sprint mechanics, and the upper body assists in counteracting the torque created by the lower body. Arm swing has been studied and proven to help the runner maintain a steady horizontal velocity [49] and counterbalance the vertical angular momentum created by the swinging legs [28,49]. A powerful upper body can assist the legs in their functions. Soccer players urge themselves forward with forceful arm drives. When they reach the maximum speed, their arms and legs collaborate to maintain the proper beat. Strong shoulders were particularly necessary. The step frequency increased automatically as the arms swung faster. Swings that are slow and lengthy have the opposite effects. This is because the brain coordinates arm and leg motions [50].

Agility and shoulder flexor and extensor strength

Soccer is a sport that necessitates high-intensity, intermittent, noncontinuous activity that incorporates agility and several sprints of varying durations [51,52]. High-speed agility accounts for approximately 11% of the total distance travelled throughout a game [1,51,53]. The ability to sprint and change direction in the face of a stimulus is a significant factor that influences performance in sports such as soccer [1,14], and the results of an agility test can better distinguish elite soccer players from the general population than any other field test [1,53]. As used in the present study, the Illinois agility test is one of the best tests for measuring soccer agility [54]. However, the present study did not find a significant correlation between agility and shoulder flexor or extensor strength. Moreover, agility also depends on the appropriate recognition of the perceptual and decision-making components and how quickly the individual responds to these, which were not assessed in the present study [55].

The present study has a few limitations. Although handheld stopwatches have proven to be reliable when measuring speed, they are not recommended when high degrees of precision are required. In addition, despite the high amount of precision maintained by the assessor and the average value of multiple readings being used, the results might have been slightly affected by the use of an HHD. Nevertheless, all testing procedures in the present study were performed using reliable instruments and standardized protocols. The study was conducted exclusively on male players, as there is limited participation of females in Indian sports [56,57]. Future studies should consider alternative strength assessment methods, such as isokinetic dynamometers, to provide more objective and reliable data. Expanding the sample to include female soccer players and individuals from diverse backgrounds would enhance the generalizability of our findings. Further studies should consider additional performance measures such as change-of-direction speed, jumping ability, and ball skills. Finally, this is a cross-sectional study and can not be used to predict the sprint performance. However, future research and randomized controlled trials might be useful in this direction.

Implications for physiotherapy practice

The findings of this study suggest that incorporating shoulder strength training into soccer players' rehabilitation and performance enhancement programs could be beneficial. Physiotherapists can prioritize shoulder strength exercises, combine them with lower-body training, tailor the training to individual needs, monitor progress and adjust training, and collaborate with coaches and trainers. By implementing these strategies, physiotherapists can help soccer players optimize their shoulder strength and improve their overall performance.

## Conclusions

The present study demonstrated a significant negative correlation between isometric shoulder muscle strength (flexors and extensors) and sprint performance in university soccer players. This study also highlighted that shoulder strength may have a positive effect on sprinting capabilities in sports. Moreover, multiple linear regression analysis also showed a positive correlation between shoulder flexor strength and sprint performance, accounting for approximately 50% of the variance. This information is useful for physiotherapists, coaches, and trainers so that they focus on strengthening the shoulder musculature to improve performance.
